# Diagnostic Yield of Ultrasound-Guided Tru-Cut Pleural Biopsy in Undiagnosed Exudative Lymphocytic Pleural Effusion in a Tertiary Healthcare Setting

**DOI:** 10.7759/cureus.95545

**Published:** 2025-10-27

**Authors:** Istikhar Ali Sajjad, Saif Ur Rahman, Usman Khalid, Huzaifa Naeem, Rubina Aman, Sarah Akram

**Affiliations:** 1 Department of Pulmonology, Allied Hospital, Faisalabad Medical University, Faisalabad, PAK; 2 Department of Pulmonology, Islamic International Medical College, Islamabad, PAK

**Keywords:** diagnostic success rate, medical thoracoscopy, pleural effusion, risks, role of ultrasound in guidance for biopsy, trucut needle

## Abstract

Background: Exudative lymphocytic pleural effusion (ELPE) has many causes including tuberculosis and malignancy. The traditional methods, such as ultrasound-guided Tru-cut pleural biopsy (UGTPB), ensure enhanced visualizations with a reduced rate of complications. The aim of this study was to establish the diagnostic yield and safety profile of UGTPB in patients with undiagnosed ELPE.

Methods: This was a prospective observational study that involved 150 patients having undiagnosed ELPE. It was conducted in a hospital in Pakistan from January to December 2024. Patients who met the light criteria of confirmed exudative, lymphocytic effusion, negative initial cytology, Ziehl-Neelsen stain, Gram stain, and cartridge-based nucleic acid amplification test (CBNAAT) were enrolled. Measures of outcomes included diagnostic yield, etiological distribution, and complications of the procedure. The statistical analysis was conducted using IBM SPSS Statistics for Windows, version 26.0 (IBM Corp., Armonk, New York, United States), and Chi-square and t-tests were used; significance level was p = 0.05.

Results: UGTPB made a conclusive diagnosis in 132 participants with an accuracy of 88%. The diagnosis of malignancy was made in 60 (40.0%) cases, tuberculosis in 52 (35.0%), and other causes in 20 (13.0%) cases. Eighteen (12.0 %) cases were not diagnosed. The sensitivity and specificity were 85% and 95%, respectively. There were three (2.0 %) patients with pneumothorax and four (2.7%) patients with minor bleeding without any major adverse events.

Conclusion: UGTPB is a low-risk, minimally invasive, and very effective diagnostic modality of ELPE. Its similarity to medical thoracoscopy with fewer complications contributes to its application as a first-line diagnostic instrument, especially in resource-limited areas.

## Introduction

Pleural effusion is a common clinical problem experienced in all parts of the world, with an approximate prevalence of 320 per 100,000 population each year [1]. Exudative pleural effusions represent a considerable percentage of these cases and are most commonly related to the diseases of tuberculosis, malignancy, or parapneumonic infections [2]. With lymphocytic and exudative pleural fluid, tuberculosis and malignancy become the two most probable etiologies [3]. Although commonly used, pleural fluid cytology can only give a conclusive diagnosis in the range of 40-60% of malignant pleural effusions [4]. Likewise, despite its historic significance, blind closed pleural biopsy has poor sensitivity and operator-dependence [5]. The constraints of these traditional methods have led to the adoption of image-guided methods for the sampling of tissues [6].

The use of ultrasound-guided pleural intervention has turned the face of diagnostic practice by not only enabling real-time visualization of abnormalities in the pleura but also reducing complications, as well as enhancing the sufficiency of the tissue [7]. Ultrasound-guided Tru-cut pleural biopsy (UGTPB) has been reported to give a diagnostic yield of 80-90% in both tuberculosis and malignant pleural effusions [8]. In addition, ultrasound is very widely accessible, inexpensive, and non-radiative, which makes it beneficial in resource-restricted environments. There is limited data on tertiary care hospitals, particularly in high-burden tuberculosis areas. In these clinical settings, an assessment of the diagnostic yield and the safety of ultrasound-guided Tru-cut pleural biopsy could provide informative evidence in the process of optimization of diagnostic algorithms and the prevention of unnecessary invasive interventions like thoracoscopy [9].

The objective of the current research was to establish the diagnostic yield and safety profile of UGTPB in patients with undiagnosed exudative lymphocytic pleural effusion (ELPE) in a tertiary healthcare facility. This evidence helped in justifying the broader use of UGTPB as a first-line diagnostic technique in high-burden areas.

## Materials and methods

This was a prospective observational study that was carried out at the Department of Pulmonology, Allied Hospital, Faisalabad, Pakistan, between January and December 2024. The study was approved by the Ethical Review Committee, Faisalabad Medical University (approval number: 48.ERC/FMU/2023-24/470). All participants provided a written consent form before enrollment, and the protocol of the study was conducted on the basis of the ethical principles of the Declaration of Helsinki.

A total of 150 sequential patients who reported undiagnosed ELPE were recruited using a non-probability consecutive sampling technique. The number of subjects was determined through the OpenEpi version 3.0.0 (2013; Centers for Disease Control and Prevention, Atlanta, Georgia, United States) by assuming a diagnostic yield of 90%, a 95% confidence interval (CI) level, a 5% margin of error, and 80% study power, which necessitated at least 139 subjects [10].

The inclusion criteria included patients aged 18 years and above with ELPE according to Light criteria, pleural fluid lymphocytosis (>50%), and a negative initial diagnostic study, including Ziehl-Neelsen stain, Gram stain, GeneXpert (Cepheid, Sunnyvale, California, United States), and cytology. Patients who exhibited transudative effusion, neutrophilic dominance, bleeding type, recent myocardial infarction, or who refused to participate were excluded.

Experienced pulmonologists who were trained in interventional ultrasound conducted an aseptic UGTPB on all patients. The localization of pleural thickening or nodularity was done by using real-time ultrasound imaging. Five to six cores of pleural tissue were collected using a 16-gauge Tru-cut biopsy needle (BARD Max Core Biopsy Needle 18 G; Becton, Dickinson and Company, Franklin Lakes, New Jersey, United States, and VELOX Tru-cut Biopsy Needle 18 G; OnkoMedica, Kiev, Ukraine) and instantly fixated in formalin and submitted to histopathology and Ziehl-Neelsen. While two to four cores are used in general, this study obtained additional cores to maximize the diagnostic tissue yield. When tuberculosis was suspected, the Xpert MTB/RIF assay (Cepheid) was carried out, following the manufacturer’s instructions. To reduce the possibility of interpretation bias, histopathologists were blinded to clinical suspicion (tuberculosis vs malignancy).

Patients have been followed up on urgent complications and had chest radiography within six hours after the procedure. To account for delayed complications, a 24-hour follow-up was also taken. The key outcome measure was diagnostic yield, which is determined as the percentage of patients who get a definite histological diagnosis. The secondary outcomes were the etiological classification (tuberculosis, malignancy, nonspecific pleuritis) and complications during the procedure (pneumothorax, bleeding, infection).

The IBM SPSS Statistics for Windows, version 26.0 (IBM Corp., Armonk, New York, United States) was used to analyze the data. Continuous variables were represented in the form of mean ± SD and were compared using a t-test. The frequencies and percentages of the categorical variables were given, and the Chi-square tests were used to compare the frequencies. Fisher’s exact test was used when expected cell counts were <5 (e.g., complication rate comparisons). A p-value of less than 0.05 was taken as significant.

## Results

A total of 150 undiagnosed patients with ELPE underwent UGTPB. Table 1 presents the baseline clinical characteristics.

**Table 1:** Baseline characteristics of participants (N=150) * = p < 0.05, χ² = Chi-square test, t value = independent t-test SD: standard deviation

Parameter	Values	Statistical Test, Value	p-value
Age (years), mean ± SD	52 ± 14.3	T value = 1.12	0.26
Male sex, n (%)	90 (60.0%)	χ²= 4.00	0.045*
Diabetes mellitus, n (%)	30 (20.0%)	χ²= 2.25	0.134
Hypertension, n (%)	45 (30.0%)	χ²= 3.18	0.075
Chronic kidney disease, n (%)	15 (10.0%)	χ²= 1.12	0.289
Smoking history, n (%)	75 (50.0%)	χ²= 5.33	0.021*

The average age of the participants was 52 ± 14.3 years, with 90 (60%) being male. The most frequent comorbidities were hypertension (n=45; 30%) and diabetes mellitus (n=30; 20%). The patients who had pleural malignancy also had a significantly higher probability of being smokers (n=75; 50.0%), in contrast to those with tuberculosis or nonspecific pleuritis (p = 0.021). Table 2 summarizes the diagnostic performance of UGTPB.

**Table 2:** Diagnostic performance of ultrasound-guided Tru-cut pleural biopsy χ² = Chi-square test; *p < 0.05 considered significant

Parameter	Value	Statistical Test, Value	p-value
Sensitivity (%)	85	χ²= 14.32	0.02*
Specificity (%)	95	χ²= 18.77	0.01*
Positive predictive value (%)	93	Fisher’s exact = 6.28	0.02*
Negative predictive value (%)	88	Fisher’s exact = 5.94	0.02*
Accuracy (%)	90	χ²= 10.21	0.03*

The findings showed that UGTPB provided high diagnostic accuracy (90%, χ² = 10.21, p = 0.003), with good specificity (95%, χ² = 18.77, p = 0.001) and sensitivity (85%, χ² = 14.32, p = 0.002). Predictive values like positive predictive value (PPV) (93%, Fisher’s exact = 6.28, p = 0.020) and negative predictive value (NPV) (88%, Fisher’s exact = 5.94, p = 0.020). The distribution of diagnostic outcomes following UGTPB was recorded as malignancy in 60 (40.0%) cases, tuberculosis in 52 (35.0%), and other pathologies, such as nonspecific pleuritis in 20 (13.0%) cases. Moreover, 18 (12.0%) cases were undiagnosed. UGTPB showed high sensitivity and specificity in relation to medical thoracoscopy, with a diagnostic accuracy of 90% in general. Table 3 provides the temporal distribution of complications.

**Table 3:** Temporal distribution of complications associated with ultrasound-guided Tru-cut pleural biopsy (N = 150) Complications assessed by chest radiography at 6 hours and clinical follow-up at 24 hours. Fisher’s exact test used for early vs delayed comparison; p < 0.05 considered statistically significant.

Complication	6 Hours, n (%)	24 Hours, n (%)	Total, n (%)
Pneumothorax	3 (2.0)	1 (0.7)	4 (2.7)
Minor bleeding	4 (2.7)	0 (0.0)	4 (2.7)
Pleural site pain	2 (1.3)	3 (2.0)	5 (3.3)
Minor infection	0 (0.0)	1 (0.7)	1 (0.7)
Major hemorrhage	0 (0.0)	0 (0.0)	0 (0.0)
Total	9 (6.0)	5 (3.3)	14 (9.3)

Complications were seen to occur in 14 patients (9.3%), all of which were minor and conservatively managed. Early complications (within six hours) were seen in nine patients (6.0%), whereas delayed complications (6-24 hours) occurred in five patients (3.3%). The difference between the early and delayed events was not statistically significant (Fisher’s exact test, p = 0.43); therefore, not mentioned in the table. Overall, UGTPB was well tolerated, and there were no serious complications.

## Discussion

This study compared the diagnostic capabilities and safety of UGTPB in patients with undiagnosed ELPE. According to our findings, the overall diagnostic yield was high, at 88% with malignancy and tuberculosis being the most common etiologies, at 60 (40%) and 52 (35), respectively. It is important to note that UGTPB was associated with an optimal safety profile and slight complications such as pneumothorax and minor bleeding. The findings indicate that UGTPB is a valuable and least invasive practice to offer a histological diagnosis of pleural effusion.

Our diagnostic result is directly comparable with other international reports where image-guided pleural biopsies were diagnosed successfully in 80-90% of cases [11]. In previous reports, UGTPB had a yield of 87%; meanwhile, a systematic review demonstrated a pooled yield of over 85% in malign and tuberculous pleural effusions [12,13]. UGTPB has a significant diagnostic benefit compared to pleural fluid cytology, which has greater than 60% sensitivity in malignant effusion [14]. The relatively equal prevalence rates of tuberculosis and malignancy among our cohort could be attributed to the dichotomous nature of infectious and oncologic disease prevalence of the South Asian population based on past studies in the area [15]. UGTPB is advantageous as it is used to provide real-time ultrasound guidance with core tissue sampling. It has been demonstrated in prior literature that blind closed pleural biopsy results in only 40-60% yields [16] and ultrasound targeting enhances the sample adequacy and lowers the complication rates [17].

The complication rate in our study was 9.3%, and no significant adverse events occurred. This relates greatly to the reported complication profile of thoracoscopy, which, despite having better sensitivity, is more invasive, has higher procedure-related morbidity, and requires more demanding equipment [18]. When put in the context of medical thoracoscopy (MTS), the findings from UGTPB were similar. It was reported that MTS had a diagnostic yield of 90-95% but was associated with increased procedural morbidity and required specialized equipment and expertise [19]. Therefore, UGTPB can be suitable in centers that lack immediate access to thoracoscopy, as it provides a viable option in making early diagnostic decisions.

The limitations of this study were that it was conducted in one tertiary care facility, and it might not be as effective as a randomized head-to-head study compared to thoracoscopy in terms of external validity. Although immediate complications were documented, long-term outcomes and delayed negative events were not examined. Thus, these findings should be confirmed by future multicenter research that should include cost-effectiveness analysis and investigate the use of advanced molecular and immunohistochemical examination of UGTPB samples. Future studies may also draw more conclusions on the usefulness of UGTPB in the initial diagnosis of the disease in high-burden facilities where tuberculosis and malignancy are the most common causes of pleural effusion.

## Conclusions

Diagnostic sensitivity of UGPB is high in patients with ELPE whose etiology was unidentified; the most frequently occurring etiologies are tuberculosis and malignancy. This was deemed to be a risk-free procedure, and there were no severe occurrences or complications that were of great concern. The results indicate that UGTPB is a valid and minimally invasive diagnostic method that potentially can promote earlier treatment and can decrease the use of more invasive diagnostic methods, including thoracoscopy.
